# Multi-Omic Biomarkers as Potential Tools for the Characterisation of Pancreatic Cystic Lesions and Cancer: Innovative Patient Data Integration

**DOI:** 10.3390/cancers13040769

**Published:** 2021-02-12

**Authors:** Laura E. Kane, Gregory S. Mellotte, Kevin C. Conlon, Barbara M. Ryan, Stephen G. Maher

**Affiliations:** 1 Department of Surgery, Trinity St. James’s Cancer Institute, Trinity Translational Medicine Institute, Trinity College Dublin, Dublin D08 W9RT, Ireland; kanela@tcd.ie; 2 Department of Gastroenterology, Tallaght University Hospital, Dublin D24 NR0A, Ireland; Gregory.Mellotte02@tuh.ie (G.S.M.); Barbara.Ryan2@tuh.ie (B.M.R.); 3 Discipline of Surgery, School of Medicine, Trinity College Dublin, Dublin D02 PN40, Ireland; kevin.conlon@tcd.ie

**Keywords:** pancreatic cancer, pancreatic cystic lesion, biomarker, risk, omics, multi-omics

## Abstract

**Simple Summary:**

Pancreatic cancer (PC) is among the most aggressive types of cancer, having caused over 495,000 deaths worldwide in 2020, with increasing annual incidence. Pancreatic cystic lesions (PCLs) are protrusions found within or on the surface of the pancreas, and in many cases have the potential to become malignant. Current methods of characterising PCLs are imperfect and there is a profound need for improved diagnostic algorithms. This review highlights the importance of biological markers in the context of PCLs and PC, with a focus on ‘omics’-related work. Successful integration of different ‘omics’ data could aid in the identification of a novel integrated biomarker profile for the risk stratification of patients with PCLs and PC.

**Abstract:**

Pancreatic cancer (PC) is regarded as one of the most lethal malignant diseases in the world, with GLOBOCAN 2020 estimates indicating that PC was responsible for almost half a million deaths worldwide in 2020. Pancreatic cystic lesions (PCLs) are fluid-filled structures found within or on the surface of the pancreas, which can either be pre-malignant or have no malignant potential. While some PCLs are found in symptomatic patients, nowadays many PCLs are found incidentally in patients undergoing cross-sectional imaging for other reasons—so called ‘incidentalomas’. Current methods of characterising PCLs are imperfect and vary hugely between institutions and countries. As such, there is a profound need for improved diagnostic algorithms. This could facilitate more accurate risk stratification of those PCLs that have malignant potential and reduce unnecessary surveillance. As PC continues to have such a poor prognosis, earlier recognition and risk stratification of PCLs may lead to better treatment protocols. This review will focus on the importance of biomarkers in the context of PCLs and PCand outline how current ‘omics’-related work could contribute to the identification of a novel integrated biomarker profile for the risk stratification of patients with PCLs and PC.

## 1. Introduction

Pancreatic cancer (PC) is the 7th leading cause of cancer-related death globally [1]. The five-year survival rate for all pancreatic cancer stages combined is just 9%, with incidence rates continuing to rise every year [2]. Pancreatic cancer can be divided into two main subtypes: pancreatic adenocarcinoma, which is responsible for 85–90% of all pancreatic neoplasms and has a five-year survival rate of just 8%, and pancreatic neuroendocrine tumour (PanNET), which is far less common and represents less than 5% of PC [1,3,4]. As pancreatic adenocarcinoma is by far the most prevalent type of pancreatic cancer, it is used synonymously with PC and will be the type of PC referred to throughout this review.

Pancreatic cystic lesions (PCLs) are typically fluid-filled structures that can be found within or on the surface of the pancreas, though some may have a solid appearance [3]. While many PCLs are benign and show no malignant potential, others, such as intraductal papillary mucinous neoplasms (IPMNs) or mucinous cystic neoplasms (MCNs), possess the ability to undergo malignant transformation and can be regarded as precursor lesions of PC [3,4,5]. The risk factors known to be associated with PC are extensive. However, investigations into these factors are largely case–control studies and as such have notable selection and recall biases [6]. Risk factors for PC can be classified as modifiable and non-modifiable [6,7]. Modifiable risk factors include lifestyle factors such as smoking and alcohol consumption, as well as conditions such as obesity [6]. Non-modifiable risk factors include age, gender, ethnicity, genetic risk factors, diabetes and chronic pancreatitis [7].

For patients who have a family history of PC or are predisposed to malignancy due to hereditary genetic mutation, PCLs can be identified in up to one-third of such high-risk individuals [8,9]. Germline mutations in *BRCA1* and *BRCA2* have been shown to confer an increased risk in PC as well as breast and ovarian cancers [10]. Von Hippel–Lindau (VHL) disease, caused by a germline mutation to the VHL tumour-suppressor gene, is associated with an increased risk of pancreatic neuroendocrine tumours and non-malignant serous-type PCLs [11].

One of the most significant risk factors for PCLs is age, with patients typically being diagnosed at 50 years or older and the incidence rate rising exponentially with age thereafter [6,7,8,12]. PCL size and number have been shown to increase with age [13]. Variations in PCL prevalence from country to country can be shown to correlate with population demographics. This geographic variance is further widened by the differences in imaging resolution and the frequency of routine physical check ups within the population [3,14]. Indeed, a 2017 study showed a positive correlation between socioeconomic development (measured through Human Development Index and Gross Domestic Product) and pancreatic cancer incidence and mortality [15]. This observed increase in PC incidence with rising socioeconomic development is thought to be result of the Western lifestyle and ageing population, which are known to be large risk factors of PC [15]. A general improvement in imaging technologies, and also a growth in the ageing population, has caused the worldwide prevalence of PCLs to rise drastically over the last two decades [8,12]. The age- and sex-adjusted prevalence of PCLs in the general population is approximately 2%, but this figure increases exponentially with age and can range up to 45% in older generations [16,17,18]. Notably, while many PCLs are found in symptomatic patients, PCLs are frequently found incidentally in patients undergoing cross-sectional imaging for other reasons—so called ‘incidentalomas’. The incidence rates of such PCLs vary depending on the imaging technique, but can be as high as 19.6% in patients undergoing magnetic resonance imaging (MRI) [19]. With the rising prevalence of PCLs globally and poor survival rates associated with PC, there is a great need for improved characterisation of pre-malignant PCLs to allow surgery in those who need it, while avoiding unnecessary surveillance and intervention in those who do not.

## 2. Current Management of PCLs

**Figure 1:**
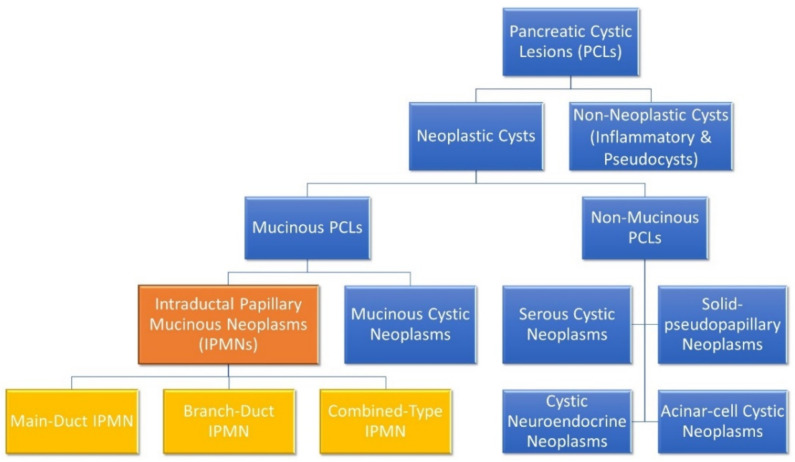
Molecular subgroups of pancreatic cystic lesions. Intraductal papillary mucinous neoplasms (IPMNs) and their distinct subclassifications are highlighted. IPMNs are the most common subgroup and are responsible for 38% of PCLs, while mucinous cystic neoplasms, serous cystic neoplasms and cystic neuroendocrine neoplasm represent 23%, 16% and 7% of PCLs, respectively [29]. Branch-duct IPMNs are most common (46%), followed by combined-type IPMNs (40%) and main-duct IPMNs (14%) [30].

When a patient with a PCL is identified, the first thing to be ascertained is its malignant potential. Broadly speaking, PCLs can be divided into either neoplastic or non-neoplastic cysts, as shown in Figure 1 [12]. Neoplastic cysts can be either mucinous or non-mucinous, with non-mucinous PCLs rarely undergoing malignant transformation [8,12]. Solid pseudopapillary neoplasms and cystic neuroendocrine neoplasms are notable, rare exceptions, as both are non-mucinous cystic lesions that do have some malignant potential and may require surgical resection. However, mucinous PCLs such as IPMNs and MCNs are generally regarded as precursor lesions for PC [8,12,20,21]. IPMNs are the most common pre-malignant PCL, being much more common than MCNs. The biological behaviour of IPMNs are notoriously unpredictable and there are currently a number of clinical guidelines that aim to help stratify the risk of IPMNs undergoing malignant transformation [22]. At present, there are three sets of guidelines in use to guide EUS and surgical referral of patients presenting with asymptomatic PCLs: the 2017 International Association of Pancreatology Fukuoka guidelines [23], the 2015 American Gastroenterological Association (AGA) guidelines [24], and the 2018 European evidence-based guidelines (EEG) [25]. The fact that there are differing consensus guidelines in use is indicative of the imperfect state of knowledge regarding PCLs and PC and the urgent need for improved biological characterisation of these lesions.

IPMNs are classified based on the involvement of the pancreatic ductal system as either main-duct (MD) IPMN, branch-duct (BD) IPMN or when both main and branch ducts are involved, combined-type IPMN [3,12]. Approximately 70% of MD-IPMNs undergo malignant transformation, whereas the rate is much lower in BD-IPMNs, ranging from 6 to 46% [3,26]. Indeed, the incidence rate of PC concomitant with BD-IPMN has been shown to range between 2% and 11.2% [27]. The World Health Organisation describes three grades of IPMN: low–intermediate-grade dysplasia; high-grade dysplasia; and IPMNs with associated invasive carcinoma [12]. Current methodologies and guidelines are limited in their ability to stratify patients into high- and low-risk of malignant transformation [28]. Identification of patients with high-grade dysplasia or early invasive cancer and the ability to predict those most likely to undergo malignant transformation is a key aspect of PCL patient management [24,28].

The most frequently used diagnostic tools for PCLs include computed tomography (CT), magnetic resonance imaging (MRI), and endoscopic ultrasound (EUS) +/- fine-needle aspiration (FNA), all of which have low sensitivity and specificity (SN/SP) for identifying high- and low-risk patients [24]. In the case of BD-IPMNs, risk stratification is based on cyst size and the presence or absence of a mural enhancing nodule. For MD-IPMNs, the diameter of the main pancreatic duct is accepted as an indicator of malignant risk [21,24,25]. A 2017 retrospective study assessed the ability of these two sets of guidelines to identify malignant cysts, and found that even when combined, 11.8% of malignant cysts were not identified [28]. Although this study had some self-identified bias (it included only higher risk patients), it highlighted the suboptimal performance of current clinical guidelines, even in expert centres [28]. Surgical resection is associated with significant morbidity and mortality and should be reserved for those at high risk of malignant transformation or established cancer [24]. Moreover, there is a 20% recurrence rate following surgical resection for IPMN [31], and recent studies have found multiple distinct regions of dysplasia within the pancreas, sometimes with differing mutational status of the same gene, supporting the notion of a multi-focal tumorigenic process of IPMN within the pancreas [4,31,32].

EUS-guided FNA is a safe and accurate method of extracting cyst fluid or pancreatic tissue from a patient for further analysis [26,33]. Cyst fluid cytology has high specificity for malignancy or high-grade dysplasia, but low sensitivity due to the typically low cellularity of PCL samples [26,33]. A 12 year multi-institutional study conducted by the French Surgical Association found that 50% of patient cyst fluid samples collected were non-diagnostic and acellular [34]. Diagnosis of PCLs by EUS requires attention to cyst morphology, including size, number of cysts present, characteristics of the wall and internal structures, calcification, positioning in relation to the main pancreatic duct and presence of lesions in the background [12]. These descriptors are considerably operator dependent and PCL characterisation without cyst fluid analysis is limited [3,26].

While the low cellularity of PCL fluid limits cytological yield, biochemical analysis of PCL fluid has proven an important adjunct in characterising PCLs. Cyst fluid CEA has been shown to have a sensitivity of between 59 and 67% and specificity of 83–91% for detection of mucinous cysts, and is among the best of the biomarkers currently available [20,35]. Mutational profiling of patients has shown utility in the characterisation of different PCL subtypes. However, genetic evaluation of PCL fluid is currently limited to research. *KRAS* and *GNAS* mutations in the cyst fluid are particularly important early mutations in IPMNs as they are not found in other common types of cysts [32]. The development of novel biomarkers within the cyst fluid has proven difficult to date due to the heterogeneic nature of the fluid. CA19-9 is a tetrasaccharide antigen released by pancreatic cancer cells and is an established marker for PC, but has low SN/SP and is not elevated in pre-malignant PCLs [36]. However, there is some evidence that CA19-9 is contributive, and most PCL guidelines advocate for its use in surveillance. Indeed, the importance of such minimally invasive serological biomarkers for use in tandem with non-invasive imaging to identify high-risk patients should not be underestimated. Based on all the aforementioned limitations of current diagnostics, it is clear therefore that there is an urgent need for novel methods and markers to accurately classify and risk stratify PCLs, and we believe that the ‘omics’ revolution is poised to fill this void of information.

## 3. Identification of Biomarkers in PCLs and PC Using Omics

The omics field has made huge strides in the past two decades, largely due to technological advancements, enabling the cost-effective and high-throughput analysis of biological molecules or ‘biomarkers’ [37]. Some omics disciplines are demonstrating great potential in the search for a novel biomarker for PC (Table 1), but the data for PCLs are much more limited.

### 3.1. Genomics

Genetic mutations have been shown to be hugely important in the study of many cancers and pancreatic cancer is no different, with *GNAS* and *KRAS* mutations representing the predominant mutations observed in this cancer [60,61]. *KRAS* is an oncogene primarily involved in the production of protein for regulating cell division [60]. Mutations in this gene are arguably the most important in the context of PC, as they frequently occur in non-cancerous precursor lesions and are subsequently present in 90–95% of all PC cases [62,63,64]. The PANDA study analysed the DNA of 113 patient cyst fluid samples in a multi-centre, prospective study [65]. Mutations in *KRAS* were shown to be indicative of a mucinous cyst, with a specificity of 96% [65]. However, there is some evidence to suggest that *KRAS* mutation alone may not be sufficient to drive a malignant phenotype, and other genetic or epigenetic events may be needed [62]. A 2011 study found that *GNAS* mutations were present in 66% of IPMNs, while mutations of either *KRAS* or *GNAS* were present in 96% [61]. The same study found *GNAS* mutations in seven out of eight cases of invasive PC that resulted from an IPMN, while it was not present in other types of pancreatic cysts or carcinoma that was not associated with an IPMN [61,66]. Mutations in both genes are believed to occur in the early stages of IPMN carcinogenesis [67]. The reported incidence of *GNAS* and *KRAS* mutations alone in IPMNs has varied greatly between studies, but a 2016 meta-analysis revealed the prevalence of *GNAS* and *KRAS* in these cysts to be 56% and 61%, respectively [68]. Simultaneous mutations in both *GNAS* and *KRAS* have been demonstrated to occur in up to 33% of IPMNs [38,69]. Indeed a *KRAS* and/or *GNAS* biomarker panel has been shown to have a SN/SP of up to 84%/98% for the identification of IPMNs (Table 1) [38,39]. Unfortunately, these results for *GNAS* and *KRAS* mutations are not mimicked in MCNs, where two separate studies revealed a SN/SP of 65%/100% for MCNs [38,40], while others have highlighted a distinct lack of *GNAS* mutations in all subtypes of MCNs [69]. Importantly, a 2019 study utilised next-generation sequencing (NGS) of formalin-fixed and paraffin-embedded tissue samples to compare mutational patterns between pancreatic ductal adenocarcinoma (PDAC) and concurrent IPMNs, identifying 3 distinct pathways by which IPMNs can progress to PDAC [70]. The ‘sequential’, ‘branch-off’ and ‘de novo’ subtypes could prove to be highly useful in the clinical characterisation and surveillance of IPMNs. Indeed, further validation of these findings and deeper exploration of the mechanisms involved is greatly needed.

Whole-genome sequencing of patients with PC, and subsequent RNAseq revealed the *KRAS* signalling pathway to be the most heavily impacted. However, further details elucidating passenger and driver mutations are needed [71]. It also appears that *KRAS* and *GNAS* mutational status vary with IPMN histological grade, adding further to the difficulties observed in these genetic mutations as potential biomarkers [69]. The feasibility of *KRAS* mutational status as a single marker has been evaluated in tissue, cyst fluid, duodenal fluid and plasma and does not appear to diagnose IPMNs or the level of cellular dysplasia consistently, being regarded as simply an early indicator of cell stress in pancreatic cells [67]. The addition of *GNAS* to pancreatic cyst fluid *KRAS* testing has been shown to increase the diagnostic accuracy of IPMN identification from 66 to 80.7%, though this does not achieve a statistically superior result to *KRAS* testing alone (*p* > 0.05), which has a diagnostic accuracy of 76.6% [39]. While mutational profiling of these genes may show some promise for IPMN identification, they provide no risk stratification for these cysts, and show little utility for MCNs compounding their lack of use in the clinical setting.

Importantly, large networks of genetic data have begun to emerge over the last two decades that contain genomic sequencing of patients with various cancer types. The Cancer Genome Atlas (TCGA) has executed the molecular profiling and subsequent analysis of over 11,000 tumours, spanning 32 different cancer types [72,73]. Tumour samples are characterised using technologies that assess the sequence of the exome, copy number variation, DNA methylation, mRNA expression and sequence, microRNA expression and transcript splice variation [73]. While this network has a substantial and diverse amount of genomic data, the matching clinical data for these patients is far more limited, and is considered to be one of the major drawbacks of the database [74,75]. The genomic data of PC patients are accessible on a number of online platforms, and several independent studies have also been launched into the utility of these data to distinguish high- and low-risk patients with PCLs. However, these single markers and panels have not yet shown sufficient sensitivity or specificity for this purpose [26].

### 3.2. Transcriptomics

MicroRNAs (miRNAs) are small, non-coding RNA molecules that function as RNA silencers and regulators of gene expression at the post-transcriptional level [76]. These molecules have been extensively studied in the context of cancer, and many miRNAs have been identified as having differential expression levels between high- and low-risk IPMNs [76]. Indeed, a 9-miRNA model developed by Matthaei et al. has shown SN/SP of 89%/100% for the distinction of high- and low-risk IPMNs by both tissue and cyst fluid (Table 1) [51]. Similarly, Lee et al. identified a 4-miRNA panel (miR-21-5p, miR-485-3p, miR-708-5p, and miR-375) that appears to distinguish IPMNs from PC, with a SN/SP of 95%/85%, though these results have not been independently validated [77]. Three long non-coding RNAs (lncRNAs) that have shown promise as risk stratification markers in the tissue of IPMN patients are CTD-2033D15.2, HAND2-AS1 and lncRNA-TFG (Table 1). A recent study conducted by Ding et al. indicated a negative correlation with tumorigenesis in IPMNs for CTD-2033D15.2 and HAND2-AS1, while a positive correlation was observed for lncRNA-TFG [41]. These results suggest a protective role of HAND2-AS1 and CTD-2033D15.2 expression in IPMNs, while lncRNA-TFG appears as a risk factor for tumorigenesis in IPMNs [41]. MiRNAs have also been identified in the blood serum and plasma of PC patients and have exhibited potential for the diagnosis of PC. Multiple studies have shown elevated levels of miR-1290 in patient blood has the ability to distinguish between PC, healthy patients, and patients with chronic pancreatitis (Table 1) [48,49,50]. Wei et al. found that miR-1290 expression was upregulated in PC patients compared to all controls, and was decreased dramatically post tumour resection (*p* < 0.001), indicating a potential role in tumorigenesis [50]. Vila-Navarro et al. used NGS to conduct genome-wide miRNA profiling and identified 30 independent miRNAs whose expression is significantly increased in PC and IPMN lesions compared to healthy individuals, and these results were validated in two independent sample sets [78]. Among these 30 miRNAs, 24 represent novel biomarkers that have not been reported previously in IPMNs [78]. While such results indicate great promise for the identification of a panel of miRNAs that could be used in pancreatic lesion characterisation, as this panel cannot distinguish IPMN from PC its clinical utility is greatly limited and larger, multi-centre studies will be needed to further interrogate and validate these results.

One limitation of current patient sampling is that EUS-FNA is an invasive procedure for patients, with sample yields often being of low volume. Research surrounding less invasive protocols has investigated the utility of salivary properties for use as non-invasive biomarkers. Salivary miRNA has been explored as a candidate for diagnostics in PC. Xie et al. validated the salivary biomarkers miR-3679-5p and miR-940 for the distinction of PC from healthy individuals, and found that combining both miRNAs produced the best discriminatory power (Table 1) [52]. Another study identified four miRNAs (miR-21, miR-23a, miR-23b and miR-29c) in patient saliva and showed them to be significantly upregulated in the saliva of PC patients when compared to healthy controls, with a sensitivity of 71.4%, 85.7%, 85,7% and 57%, respectively, and specificity at a fixed 100% [79]. However, these same miRNAs were shown to be detected in patients with pancreatitis, while miR-23a and miR-23b were detected in patients diagnosed with IPMNs [79]. While these miRNAs show promise in distinguishing PC from healthy controls, as patients with pancreatitis and precursor lesions have also been shown to express these markers, further validation is required on a larger, external cohort to fully demonstrate the utility of these miRNAs at distinguishing various pancreatic pathologies. A 2020 systematic review and meta-analysis interrogated the potential of various salivary biomarkers in several cancer types, including transcriptomic, epigenomic (*v. inf.)* and microbiomic markers in PC, and found “good” diagnostic accuracy for such markers in PC with an area under the curve (AUC) of 0.87 [80]. However, the review also highlighted the high degree of variation in the sensitivity (31–100%) and specificity (34–100%) observed in different studies of salivary biomarkers in non-oral cancers, and further interrogation of the data revealed that the probability of a patient having some malignancy is 31% if the salivary test result is negative [80]. While investigations into the use of salivary properties for diagnostic and prognostic purposes appears promising, further work is required to identify more robust biomarkers.

Transcriptomic data, as highlighted above, has profound potential for the identification of a novel biomarkers. Compilations of transcriptomic data can be found readily available in online repositories such as the Gene Expression Omnibus and EBI ArrayExpress [81,82]. It is evident from the expansive transcriptomic data available for PC that much research has been conducted. However, to date, no transcriptomic biomarker among the many identified has been approved for use in this cancer. While vast quantities of data are often favourable, it appears that more information and progress may be gained from the integration of different data types.

### 3.3. Epigenomics

A histone methyltransferase known as enhancer of zeste homologue 2 (EZH2) is known to be overexpressed in many cancers, including PC, and has also been detected in IPMNs with moderate to severe dysplasia [83]. A 2010 study showed that high expression levels of EZH2 in PC were associated with increased node positivity and a larger tumour size; EZH2 expression levels were also shown to relate to the degree of dysplasia in IPMNs [83]. RNA interference silencing of EZH2 sensitised PC patients to treatment with gemcitabine, resulting in significantly longer overall survival [83]. Such RNA interference silencing of EZH2 has been utilised in a PC model and caused a decrease in tumour growth and the incidence of liver metastasis [84]. More recent investigation into EZH2 has highlighted its role in the epigenetic repression of tumour-suppressor gene expression. Trimethylation of *H3K27* by EZH2 allows the mediation of cell proliferation, invasion and migration [85]. Exposure of F-box and WD repeat domain-containing 7 (FBW7) to EZH2 causes the degradation of EXH2 in PC cells and inhibits tumour migration and invasion, indicating its role as a ligase of EZH2 that regulates EZH2 protein levels in PC and furthermore, its potential as a treatment strategy [85]. Indeed as epigenetic alterations are reversible and plastic, they can be regarded as more amenable to therapeutic intervention than non-reversible genetic mutations [86]. Hata et al. identified 6 methylated DNA markers in patient cyst fluid that could distinguish high- and low-risk PCLs with accuracies from 79.8 to 83.6% [46]. Methylated *SOX17* was shown to be the most sensitive single marker, while a four-gene combination (*FOXE1*, *SLIT2*, *EYA4*, *SFRP1*) showed the highest accuracy at 88% (Table 1) [46]. Furthermore, cyst fluid obtained from IPMNs with high-grade dysplasia had significantly higher levels of methylated DNA than other mucinous cysts [46]. A more recent study by Eissa et al. examined the cell-free DNA in patient blood, and found the methylated gene of *ADAMTS1* to have a SN/SP of 87.22%/95.8% for the differentiation of PC and normal samples [45]. Moreover, the same study showed that the addition of a second methylated gene, *BNC1*, such that either or both were detected in the samples, showed even better SN/SP for the same purpose (Table 1) [45].

For IPMNs, the epigenetic data currently available is limited. One gene whose promoter is known to be hypermethylated in almost all cancer types is the *cysteine dioxygenase 1* (*CDO1*) gene. A recent study examined this gene in pancreatic IPMN tumour tissue and found the *CDO1* promoter hypermethylation is extremely specific to IPMNs and appears to accumulate with tumour progression [87]. Among other pancreatic disease, low levels of *CDO1* promoter hypermethylation were seen in MCNs, with no other pancreatic cystic disease showing DNA hypermethylation of its promoter. A pilot study in pancreatic juice confirmed methylation in all IPMN samples (*n* = 6) with none detected in benign pancreatic diseases (*n* = 6, chronic pancreatitis and autoimmune pancreatitis) [87]. Furthermore, *CDO1* hypermethylation showed utility in the differentiation of low–intermediate-grade dysplasia and high-grade dysplasia/PC [87]. While these results show promise in the search for a biomarker to stratify IPMN patients, extremely robust thresholds for *CDO1* methylation are needed to distinguish high- and low-risk patients, with little utility being seen for other PCLs. Further analyses on a large patient cohort, examining the methylation status of *CDO1* in patients with pancreatitis, pseudocysts and a variety of pancreatic cystic lesions would be required to further validate this marker.

The establishment of epigenomic databases such as ENCODE, The International Human Epigenome Consortium and Roadmap Epigenomic Project, has enabled the popularisation of epigenomics and allowed for the establishment of standardised sequencing methods [88]. Furthermore, epigenome-wide association studies (EWAS) in combination with GWAS and TWAS data have proved to be powerful tools in pinpointing disease-relevant regulatory elements [88,89].

### 3.4. Proteomics

The proteome can be examined at different developmental or cellular phases, and changes in the proteome can be evaluated at different time points. Proteomics can be a qualitative and/or quantitative evaluation of the proteome and is generally conducted using mass spectroscopy (MS) [90,91,92]. As mentioned previously, two markers currently utilised in PC are CA19-9 and CEA, and these markers have been shown in many instances to be insufficient in the discernment of IPMNs and MCNs, as well as their malignant potential (Table 1) [93,94,95]. CEA levels in patient cyst fluid can be used to distinguish between mucinous and non-mucinous cysts, but have limited sensitivity (58–73%) and specificity (89–96%) [42]. Kadayifci et al. evaluated the diagnostic accuracy of adding CEA to the *KRAS* and/or *GNAS* panel but found it did not provide better SN/SP (*p* > 0.05) than the *KRAS* and/or *GNAS* panel alone [39,43]. Indeed, a recent study showed that artificial intelligence by deep learning has better SN/SP (95.7/91.9%) for diagnosis of malignant cystic lesions than CEA levels and cytologic analyses [95]. Serum CA19-9 is the only FDA-approved marker for the identification of PC. However, it has been demonstrated that CA19-9 alone failed to detect 44.1% of cancer cases in a cohort of 34 patient samples, and added no improvement to the sensitivity of the two-gene methylation panel *ADAMTS1* and/or *BNC1* [45]. CA19-9 is widely regarded as not sufficiently sensitive to distinguish PC from healthy samples as it is frequently elevated in non-malignant conditions such as pancreatitis, and has been shown to have a SN/SP of 52.7%/90% [53]. However, the addition of CA19-9 to a marker panel (CA19-9, ICAM-1, OPG) was shown to produce better sensitivity and specificity for PC (78% and 94.1%, respectively) [53]. Indeed, Brand et al. identified several 3-marker panels that offered an improved ability over CA19-9 alone to distinguish PC from healthy controls [53]. One multi-institutional group has shown in multiple cohorts that the combination of CA19-9 and apolipoprotein-A2 isoforms can improve the diagnostic ability of CA19-9 alone in the detection of PC up to 18 months prior to diagnosis under typical clinical conditions [96,97,98]. Another study identified how the change in cut-off value for CA19-9 can improve the robustness of this marker, but also showed that the addition of CA19-9 to a marker panel gave the best SN/SP when compared to CA19-9 alone (*p* < 0.05) [54]. While CA19-9 appears to have limited use clinically for diagnostic screening of patients for PC, it does have utility in predicting disease recurrence post-treatment [55].

The identification of novel protein markers in PC has been of great interest over the last two decades, as those markers in current clinical use are imperfect. The protein component of pancreatic cyst fluid has not yet been well characterised, as interrogation of the proteome is relatively new and technological advances are frequently being made [3]. Individual proteins such as thymosin-β4 and ubiquitin have been found to be significantly overexpressed in the tissue of IPMNs with high-grade dysplasia (*p* = 0.011 and 0.04, respectively) [59]. Panels of proteins have also shown promise for the differentiation of mucinous and non-mucinous cysts. Elevations in any two of the 3-protein panel MUC5AC:WGA, MUC5AC:BGH and Endorepellin:WGA has shown good SN/SP for the identification of MCNs (Table 1) [57,58]. Porterfield et al. utilised proteomic analysis by liquid chromatography-MS to identify seven proteins shown to be consistently increased in the ductal fluid of PC patients compared to normal (AMYP, PRSS1, GP2-1, CCDC132, REG1A, REG1B, and REG3A), as well as one that was decreased (LIPR2), and validated these results by Western blot [99]. A recent meta-analysis combined publicly available proteome and secretome data with the aim of identifying biomarkers of PC. While this analysis did not identify any protein that was shared by all of the 55 included secretome and proteome studies, by selecting proteins found in 2 or more studies an intersection between the two exposed 43 proteins common between proteome and secretome analyses [100]. Notably, 31 genes related to these secretome-proteins were shown to be upregulated in PC samples obtained from TCGA compared to control samples, while 39 such genes were revealed to be predictors of worse overall survival in PC [100].

As IPMNs are classified as mucinous cysts, it follows that their composition is partially composed of mucin proteins. Mucins, which are densely O-linked glycoproteins with a high molecular weight, play many roles in the maintenance of pancreatic health and subsequently, when altered as a result of malignancy can be important facilitators of tumorigenicity [67]. IPMNs are known to have a unique pattern of mucin expression, and this trait has been utilised in the subclassification of IPMNs [101]. Indeed, mucin proteins have been extensively investigated in the context of mucinous PCLs and evaluated as potential biomarkers but to largely no avail [67,92,101] Moreover, while many studies have examined the mucin proteins of the cyst type, surprisingly, research has shown no significant pattern of RNA expression of mucin proteins identified in IPMNs [102]. The combination of mucin proteins into panels, as mentioned previously, has shown promise in the distinction of mucinous and non-mucinous cysts with good SN/SP [57,58]. Though not a mucin, VEGF-A is also a glycoprotein and is known to be a key mediator of vascular growth [103]. Elevated levels of VEGF-A have been observed to indicate the presence of a benign serous cystic neoplasms with high SN/SP (Table 1) [56,103]. Furthermore, the addition of CEA levels to VEGF-A exhibited better still SN/SP for the identification of these cysts [56].

An important aspect of proteomic work in mucinous PCLs is the depletion of larger proteins, which in this case is not just IgG and albumin, but also the mucin proteins. The exclusion of larger, more abundant proteins by such immunodepletion steps increases assay sensitivity for smaller proteins that may not otherwise be detected. Depletion based on molecular weight is frequently employed. However, the purity of the protein samples obtained by this method is generally poor. Indeed, the discovery of mucin-specific proteases that could aid depletion of these proteins appears to be making strides. A recent study by Malaker et al. identified a mucin-selective protease, StcE, which shows great promise in the selective digestion of human mucins from biological samples [104]. Interestingly, a recent study examined the protein component of IPMN cyst fluid supernatant and cell pellet, reporting that the cell pellet contains twice as many proteins as the supernatant and even contained over two thousand that were not identified in the supernatant [3]. This study opted to omit the immunodepletion step that is routinely used in proteomic analyses, and in doing so identified almost 4000 proteins previously unknown in pancreatic cyst fluid [3]. This large, proteomic dataset has been deposited into the ProteomeXchange database, with the hope that it may prove a rich source of information for further IPMN studies [3]. Other online platforms of proteomic data, such as the Clinical Proteomic Tumour Analysis Consortium and PRoteomics IDEntification database allow users to upload their own data or examine those datasets submitted by others to supplement new research [105,106].

Often overlooked in proteomic studies is the part that genomic changes play in the alteration of the proteome. A 2014 study integrated proteomic and TCGA data for colon and rectal tumours, and found that messenger RNA transcript abundance did not correspond with the difference in protein abundance observed between tumours [107]. Similar research conducted in 2016 showed how the integration of proteomic and phosphoproteomic analyses with TCGA data for 77 genomically annotated breast cancers enabled the discovery of novel functional consequences of somatic mutations in this cancer type, and subsequently narrowed the scope of potential candidates for driver genes [108].

### 3.5. Metabolomics

Metabolomic alteration of cancer cells has been regarded for nearly a century as one of the hallmarks of cancer [109]. Indeed, the switch of metabolic pathways observed in cancer cells is regarded as key for tumour growth, and is suspected to be selected for during transformation [109]. Recent studies conducted by Mayerle et al. identified a biomarker signature of nine metabolites alongside CA19-9 in the blood using mass spectrometry (MS) for the distinction of PC and chronic pancreatitis [110]. While not validated, this study showed the potential of this panel in both a training and test cohort, with SN/SP of 89.9%/91.3% [110]. Fahrmann et al. also utilised MS for the identification of a metabolite panel in the blood [44]. This panel was observed to distinguish PC from normal samples with moderate SN/SP (Table 1) [44]. Metabolic profiling combining MS and liquid chromatography techniques enabled the discovery of 55 metabolites that were differentially expressed in pancreatic tumours as compared to non-tumours (*p* < 0.01) [111]. Further examination of these metabolites using weighted co-expression network analysis highlighted eight fatty acid hubs that are highly connected and in a conserved lipid module that are decreased in PC tumours compared to the surrounding non-tumour tissue [111]. Integration of transcriptomic data revealed 157 gene surrogates for this fatty acid set and showed that the expected lipid metabolism, particularly in the lipolytic pathway involving these gene surrogates, is significantly altered in PC [111]. These data suggest a dysregulation of the lipolytic network in PC which may play some role in tumorigenesis. Kynurenine, a metabolite known to be synthesised in response to immune activation, has shown promising ability to discern mucinous from non-mucinous PCLs with high SN/SP 90%/100% [42]. This metabolite, is surprisingly detected in lower levels in the cyst fluid of MCNs compared to non-MCNs, suggesting some dampening of immune activation in MCNs. In that same study, Park et al. identified 10 metabolites that were differentially abundant in their validation cohort, 8 of which could not be matched to any known metabolite and mass spectrometry analysis was unsuccessful due to the low abundance [42]. Importantly, glucose levels in the cyst fluid have also been observed to discriminate MCNs from non-MCNs, and a standard patient glucometer has been shown successful in this manner [42,43]. If cystic glucose levels could be correlated to same in patient blood samples, this methodology could prove a less invasive manner of determining cyst type. However, such correlations would be highly unlikely given the plethora of factors which can influence blood glucose levels, such as the presence of diabetes and patient fasting status.

As metabolomics is a relatively new field of study, there is little research performed in the context of PC. The establishment of large metabolic databases, such as the Metabolomics Workbench, could enable large metabolic studies in PC and subsequent integration of this information with other omics data to produce a robust biomarker panel [112].

## 4. Multi-Omics as the Key to Biomarker Identification

In the case of PCL and PC characterisation, though these new ‘omics’ techniques have been utilised to analyse the pancreatic cyst fluid, blood serum and even saliva of patients, no single methodology has proven to be a sufficiently sensitive method for delineating these patients into defined categories. Multi-omics involves the integration of multiple layers of omics-type data to augment our understanding of disease and helps researchers to elucidate the flow of information, from the origin of the disease to the biological and functional consequences [37]. By investigating multiple aspects of the PCL fluid or blood serum, and treating these data as an interconnected system, rather than distinct and independent pieces, multi-omics could allow researchers to identify key pathways and players in disease stratification. CompCyst is a comprehensive test developed using machine learning techniques to guide the management of patients with PCLs [113]. This test utilises selected clinical features such as symptoms, cyst size and location, as well as cyst fluid genetic and biochemical markers, including cyst CEA levels and *KRAS* and *GNAS* mutation status [113]. Interrogation of multiple levels of patient data enabled cut offs for each marker to be determined based on the needs of the test, and the level of importance given to the sensitivity or specificity of each individual marker. The results of this study suggest that if CompCyst were applied to general PCL management, 60% of unnecessary surgeries could be avoided [113]. While these results seem promising, it is important to note that patients evaluated in this study were those most concerning for cancer and do not represent patients seen in routine clinical practice [113]. While more research is needed to examine the utility of this test in a normal clinical setting, this study shows that layering multiple levels of patient data can potentially improve management strategies for PCLs. Interestingly, a 2018 report describes a multi-analyte blood test called CancerSeek, which assesses levels of circulating proteins and mutations in cell-free DNA to detect one of eight common cancer types (ovarian, liver, stomach, pancreatic, oesophageal, colorectal, lung, or breast) [114]. When combined with supervised machine learning, this test was able to localise the source of the cancer to two anatomic sites in a median of 83% of patients (*n* = 626). While not specific to PC, this test shows the stark advantage of combining distinct approaches to create a robust diagnostic tool.

In terms of PCLs and PC, multi-omics opens the door to the possibility of a biomarker panel for characterisation, such that combined thresholds of several markers could prove more sensitive than a single marker alone. A 2020 systematic review examined novel biomarkers for upper GI cancers, identifying 431 biomarkers, of which more than half (*n* = 231) were for PC [115]. Only one-fifth of the biomarkers reported in this review were examined in more than one study, and of those that were, there were only two single markers and one panel of markers for PC. Such reviews of the literature show the current state of PC research, where most of those markers that are identified are not examined further and as such never become clinically useful. As mentioned previously, the addition of *GNAS* to *KRAS* testing for the diagnosis of IPMNs does not significantly increase diagnostic accuracy. However, the same study found that the combination of *GNAS* and *KRAS* mutational status with CEA testing does produce a significantly better accuracy of 86.2% (*p* < 0.05) [39]. A 2015 multi-centre study retrospectively examined the cyst fluid of 130 patients and identified molecular markers and clinical features that classified PCLs with a sensitivity of 90–100% and a specificity of 92–98% [116]. Using the Multivariate Organisation of Combinatorial Alterations (MOCA) algorithm to identify composite clinical and molecular markers (subtle mutations, loss-of-heterozygosity, aneuploidy) of PCL type and grade, this study identified a panel of both clinical and molecular markers for the distinction of serous cystadenomas (SCA), solid-pseudopapillary neoplasm (SPN), MCNs and IPMNs. Furthermore, it was shown that these features could identify 67 of the 74 patients who did not require surgery, resulting in a reduction in unnecessary procedures by 91%. These results show great promise for the characterisation of PCLs and the stratification of patients for subsequent referral to surgery, and further studies in more robust, experimental validation cohorts will help to further elucidate the potential of this panel in the context of PC.

**Figure 2:**
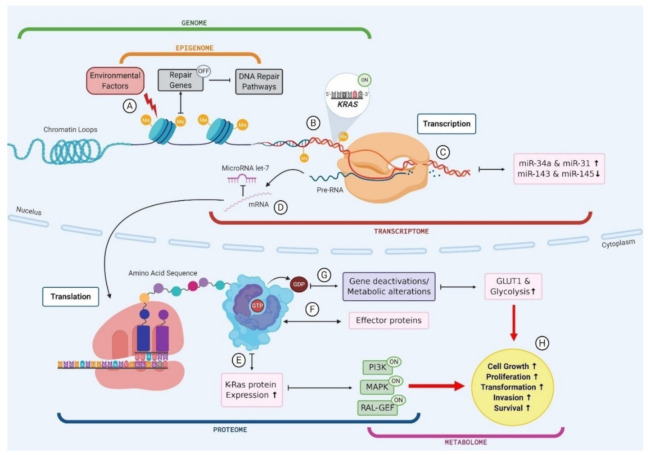
The multi-omic nature of KRAS mutations in pancreatic cancer. (**A**) Environmental factors cause biochemical alterations to the DNA such as hypermethylation. This can result in the silencing of repair genes and subsequently failure in DNA repair pathways; (**B**) Point mutations in a KRAS codon go unchecked as a result of DNA repair failure causing permanent activation of KRAS gene; (**C**) Mutant KRAS gene is transcribed into mRNA and subsequently results in an upregulation of miR-34a and miR-31 and a downregulation in miR143 and miR-145; (**D**) mutant KRAS mRNA cannot be bound by regulatory miR let-7 and leaves the cell nucleus to be translated; (**E**) Mutant KRAS causes an increase in K-Ras protein expression, which causes activation of PI3K, MAPK and RAL-GEF pathways; (**F**) GTP bound KRAS interacts with various effector proteins and influences the localisation and activities of these effectors; (**G**) K-Ras proteins convert GTP to GDP which causes gene deactivations and metabolic alterations such as an increase in GLUT1 expression and subsequently an increase in glucose uptake via glycolysis; (**H**) Changes to cellular protein expression, gene activation and metabolic processes results in increased cell growth and proliferation, driving transformation.

A key example of the multi-omic nature of driver mutations in the context of PC is *KRAS*, which is mutated in ~90% of PC (Figure 2) [117]. Environmental factors, such as smoking or alcohol consumption, can promote biochemical alterations to DNA at the epigenomic level, for example hypermethylation [118]. The addition of a methyl group to the CpG island of a DNA repair gene can cause silencing and subsequently result in reduced DNA repair proficiency, allowing a mutated *KRAS* codon to proceed from the genomic level to the transcriptomic level. The transcription of this *KRAS* mutation results in altered miRNA expression levels, and the mutated mRNA cannot be bound by the regulatory miRNA let-7, thus causing the aberrant translation of K-Ras protein [119,120,121]. The marked increase in K-Ras production promotes various signalling pathways, including phosphoinositide 3-kinase (PI3K), mitogen-activated protein kinase (MAPK) and the RAL-GEFs pathway [117]. GTP-bound K-Ras proteins can interact with, and influence the activity of, effector proteins causing downstream effects in many cellular pathways [117]. Moreover, *KRAS* mutant PCLs have been shown to have increased expression of the glucose transporter GLUT1 and subsequently elevated rates of glycolysis, indicating that *KRAS* mutations play a role in the metabolic switch observed in PC [122]. Indeed, the presence of *KRAS* mutations in PC has been shown to correlate with poor patient prognosis, and this can be attributed to the downstream effects seen in multiple omics layers as a result of this point mutation (Figure 2) [123]. This example illustrates the multi-omic nature of mutational drivers in cancer and the importance of disentangling each aspect in order to clearly observe the pathways affected and the impacts at each omics level.

As discussed above, each omics discipline has its own advantages and disadvantages, and can give information about many aspects of disease from metabolic signatures to proteomic profiles. It is only logical therefore to examine this extensive information in parallel with the aim of revealing those attributes that can be considered robust and sensitive enough to work as a biomarker of patient risk. Typical analysis of a single omics data type is largely limited to correlations and tends to reflect the reactive processes of disease, rather than the causative [37]. However, compilation of many data types can enable statisticians to tease out the causative and resultant factors observed in these data by enhancing the statistical depth and power of the dataset. In this way, sufficient statistical power obtained by having a large cohort is required for any successful omics study, in order to produce the most robust results [37].

The LinkedOmics database contains multi-omics data within and across 32 cancer types for over 11,000 patients from TCGA [72]. This platform is the first of its kind, and integrates data generated by the CPTAC for select TCGA tumour samples and has therefore, over a billion data points [72]. The database allows users to apply comprehensive analyses on these data by use of three distinct modules: LinkFinder, which identifies associations between clinical and molecular attributes of interest; LinkCompare, which enables comparisons of those associations obtained via LinkFinder; and LinkInterpreter, where identified associations are further explored through pathway and network analysis. Through the use of several case studies, examining properties of individual cancers to reveal functional impacts of somatic mutations or copy number alteration on the expression of mRNA and protein, or performing pan-cancer analysis to investigate survival-associated gene expression signatures, the power of such multi-omics platforms can be seen [72]. While this database only includes data from TCGA and CPTAC, the extension of its data collection for more cancer types and omics platforms could enable the execution of robust and highly powered multi-omics studies for many cancer types. The TCGA data were utilised in a recent multi-omics study of PC, where the integration of DNA copy number variation, methylation, mRNA, and simple nucleotide variation data enabled the identification of four distinct molecular subgroups of PC (iC1, iC2, iC3, and iC4) [124]. The iC1 subgroup was shown to have a better prognosis, higher immune cell infiltration and better genomic stability compared to the other groups. Furthermore, this multi-omics study identified three new genes (*GRAP2*, *ICAM3* and *A2ML1*) that were shown to correlate with prognosis in PC. A 2019 study utilised a multi-omics approach comprising exome sequencing, transcriptomics, quantitative proteomics, karyotyping and metabolic status to evaluate the epithelial-mesenchymal plasticity of two sister breast cancer cell lines, identifying novel driver mutations, chromosomal changes, gene deletions/ amplifications, alterations in gene expression and metabolic reprogramming [125].

With the exception of somatic mutations, the original human DNA sequence is unaltered throughout life, being unaffected by environmental or developmental factors [37]. As such, it is generally assumed that any disease-related genetic mutation observed is causal and not a result of disease. This assumption is often the reason for the level of difficulty attributed to distinguishing the causative agent of disease from those effects that are created as a result of disease [37]. The tumour profiles of clinically identical patients have been observed to share as few as one single genetic mutation [126]. In the context of PC, the integration of data from multiple types of omics can help to reveal the biochemical pathways involved and ultimately those genes that are playing an active role in tumour growth. This layering of data in a multi-omics approach can help to tease out such details and begin to show the picture in its entirety, not only indicating the causative agent, but also the downstream pathways and interactions involved.

## 5. Conclusions

PC is an aggressive disease with extremely poor survival rates. The discovery of precursor lesions often occurs too late, and patients are left with few treatment options. PCLs are a highly diverse group of lesions containing both non-malignant and pre-malignant subtypes, and there exists no robust method for distinguishing PCLs and subsequently, which patients have a high-risk of developing PC and should undergo surgical resection, and which patients are at a lower risk and can be spared this procedure. The advent of omics has enabled significant strides in the detection and treatment of cancer. Unfortunately, for patients with PCLs or PC, current individualised omics studies have produced little success. Multi-omics provides a more comprehensive insight into the mechanisms and pathways involved in cancer and has great potential for use in diagnosis and treatment of PC. The presence of many omics databases online, which are publicly available and contain vast quantities of patient data, enables the interrogation of large datasets and the production of highly powered studies. However, there are many facets of the integration of data that must be acknowledged and sufficiently managed in order for these studies to produce accurate and robust results. There is a need for standardisation of multi-omics approaches in this way, such that more in-depth analyses can be carried out. The discipline of multi-omics imparts much expectation for the further understanding of PC as a disease, and the identification of biological markers that may aid in the characterisation of patient PCLs.

## Figures and Tables

**Table 1:** Overview of biomarkers in PC and PCLs that have been validated in an independent cohort.

Biomarker Name	Biomarker Type	Single or Multi-Study Validated	Platform	Sample Type	Sample Size (Total Number Patients)	Sensitivity (95% Confidence Interval)	Specificity (95% Confidence Interval)	P-Value	Purpose	References
KRAS and/OR GNAS	Genetic mutation panel	Multi	PCR	Cyst fluid	91	65%	100% (83–100)	N/A	MCN vs. non-MCN	[38]
						(52–76)	98%			
						84%	(86–100)	N/A	IPMN vs. non-IPMN	
						(70–92)				
							95.50%			
			PCR using NGS	Cyst fluid	197	68.50%	(N/A)	N/A	IPMN vs. non-IPMN	[39]
						(N/A)				
							100%			
			NGS	Cyst fluid	595	89%	(88–100)	N/A	MCN vs. non-MCN	[40]
						(79–95)	100%			
			Sanger sequencing	Cyst fluid	159	65%	(N/A)	N/A	MCN vs. non-MCN	
						(N/A)				
lncRNA-TFG	Long noncoding RNA	Single	Affymetrix human exon 1.0 ST	Tissue	28	N/A	N/A	6.23 × 10^−8^	Positive correlation with tumorigenesis in IPMNs	[41]
CTD-2033D15.2	Long non-coding RNA	Single	Affymetrix human exon 1.0 ST	Tissue	28	N/A	N/A	1.47 × 10^−4^	Negative correlation with tumorigenesis in IPMNs	[41]
HAND2-AS1	Long non-coding RNA	Single	Affymetrix human exon 1.0 ST	Tissue	28	N/A	N/A	2.66 × 10^−3^	Negative correlation with tumorigenesis in IPMNs	[41]
Glucose	Metabolite	Multi	Liquid chromatography	Cyst fluid	19	94%	64%	0.004	Glucose ≤ 66 mg/dL in MCNs vs. non-MCNs	[42]
						(N/A)	(N/A)			
			Glucometer						Glucose ≤ 50 mg/dL in MCNs vs. non-MCNs	
				Cyst fluid	153	92%	87%	N/A		[43]
						(N/A)	(N/A)			
Kynurenine	Metabolite	Single	Liquid chromatography	Cyst fluid	19	90%	100%	0.002	Lower in MCNs vs. non-MCNs	[42]
						(N/A)	(N/A)			
AcSperm and	Metabolite panel	Single	Mass spectrometry	Blood plasma	121	66.70%	95%	N/A	PDAC vs. N	[44]
DAS and						(N/A)	(N/A)			
LPC(18: 0) and LPC(20: 3) and indole derivative										
ADAMTS1	Methylated gene	Single	Methylation on beads	Blood cfDNA	39	87.20%	95.80%	N/A	PDAC vs. N	[45]
						(N/A)	(N/A)			
BNC1	Methylated gene	Single	Methylation on beads	Blood cfDNA	39	64.10%	93.70%	N/A	PDAC vs. N	[45]
						(N/A)	(N/A)			
SOX17	Methylated gene	Single	Methylation-specific ddPCR	Cyst fluid	154	78.40%	85.60%	N/A	High-risk PCL vs. low-risk PCLs	[46]
						(64.7–88.7)	(78.4–91.1)			
TBX15 and BMP3	Methylated gene marker panel	Single	Whole-genome methylome discovery and qPCR	Cyst fluid	134	90%	92%	N/A	HGD/PC vs. LGD/N	[47]
						(70–99)	(85–96)			
ADAMTS1 and/OR BNC1	Methylated gene panel	Single	Methylation on beads	Blood cfDNA	39	97.40%	91.60%	N/A	PDAC vs. N	[45]
						(N/A)	(N/A)			
FOXE1 and SLIT2 and	Methylated gene panel	Single	Methylation-specific ddPCR	Cyst fluid	154	84.30%	89.40%	N/A	High-risk PCL vs. low-risk PCLs	[46]
EYA4 and SFRP1						(N/A)	(N/A)			
miR-1290	MicroRNA	Multi	MicroRNA array analysis	Blood serum	60	88%	84%	N/A	PC vs. N	[48]
						(N/A)	(N/A)			
					76	83%	69%	N/A	PC vs. CP	
						(N/A)	(N/A)			
					95	83%	78%	N/A	PC vs. CP and N	
						(N/A)	(N/A)			
			qRT-PCR	Blood plasma						
					49	N/A	N/A	0.027	PDAC vs. N	[49]
				Blood serum						
			qRT-PCR							
					200	74.20%	91.20%	N/A	PC vs. C	[50]
						(N/A)	(N/A)			
9-miRNA model ^a^	MicroRNA panel	Single	TaqMan miRNA Array	Tissue and	33 and 50	89%	100%	N/A	HG IPMNs, PanNETs and SPNs vs. LG IPMNs and SCAs	[51]
				cyst fluid		(N/A)	(N/A)			
miR-3679-5p and miR-940	MicroRNA panel	Single	qPCR	Saliva	80	72.50%	70.00%	N/A	PC vs. N	[52]
						(N/A)	(N/A)			
					60	62.50%	80.00%	N/A	PC vs. BPT	
						(N/A)	(N/A)			
					100	70.00%	70.00%	N/A	PC vs. N and BPT	
						(N/A)	(N/A)			
CA19-9	Protein-	Multi	Bead-based xMAP immunoassay	Blood serum	267	57.20%	90%	N/A	PDAC vs. N	[53]
	associated					(N/A)	(N/A)			
			ELISA							
				Blood plasma						
					176	77.50%	83.10%	N/A	CA19-9 >20.3 U/mL	[54]
			Retrospective clinical data	Blood serum		(N/A)	(N/A)		PDAC vs. C	
					41	90%	83.33%	N/A	2.45 times elevated CA19-9 indicated recurrence of PC	[55]
						(N/A)	(N/A)			
CEA	Protein	Multi	Clinical data	Cyst fluid	31	73%	89%	N/A	CEA > 192 ;ng/mL for MCN	[42]
						(N/A)	(N/A)			
			ELISA	Cyst fluid	149	95.50%	81.50%	<0.0001	CEA ≤ 10 ng/mL for SCN	[56]
						(N/A)	(N/A)			
			Enzyme-linked immunosorbent assay	Cyst fluid	153	58%	96%	N/A	CEA > 192 ng/mL for MC	[43]
						(N/A)	(N/A)			
MUC5AC:WGA and MUC5AC:BGH and Endorepellin:WGA	Protein	Multi	Antibody-lectin sandwich microarray	Cyst fluid	147	92% ^b^	94% ^b^	N/A	Elevation in any two differentiates MCNs vs. non-MCNs	[57]
	panel					(N/A)	(N/A)			
			Antibody-lectin sandwich arrays						Elevation in any two differentiates MCNs vs. non-MCNs	
				Cyst fluid	22	87%	100%	N/A		[58]
						(N/A)	(N/A)			
Thymosin- β4	Protein	Single	MALDI imaging and mass spectrometry	Tissue	45	70%	71%	0.011	Overexpressed in IPMN with HGD	[59]
						(N/A)	(N/A)			
Ubiquitin	Protein	Single	MALDI imaging and mass spectrometry	Tissue	45	94%	86%	0.04	Overexpressed in IPMN with HGD	[59]
						(N/A)	(N/A)			
VEGF-A	Protein	Single	ELISA	Cyst fluid	149	100%	83.70%	<0.0001	VEGF-A > 5000 pg/mL benign SCN	[56]
						(N/A)	(N/A)			
VEGF-A and CEA	Protein panel	Single	ELISA	Cyst fluid	149	95.50%	100%	N/A	VEGF-A > 5000 pg/mL and CEA ≤ 10 ng/mL in benign SCN	[56]
						(N/A)	(N/A)			

BPT = benign pancreatic tumour, C = non-cancer control, CP = chronic pancreatitis, ELISA = enzyme-linked immunosorbent assay, HG = high grade, HGD = high-grade dysplasia, IPMN = intraductal papillary mucinous neoplasm, LG = low grade, LGD = low-grade dysplasia, MALDI = matrix-assisted laser desorption ionisation, MC = mucinous cyst, MCN = mucinous cystic neoplasm, N = normal healthy, N/A = not available, NGS = next-generation sequencing, PanNET = pancreatic neuroendocrine tumour, PC = pancreatic cancer, PCL = pancreatic cystic lesion, PCR = polymerase chain reaction, PDAC = pancreatic ductal adenocarcinoma, SCN = serous cystic neoplasm, and SPN = solid-pseudopapillary neoplasm. ^a^ Model is intellectual property of the authors. ^b^ Average of three cohorts.
